# Comparison of the NIST and BIPM Medium-Energy X-Ray Air-Kerma Measurements

**DOI:** 10.6028/jres.108.032

**Published:** 2003-10-01

**Authors:** D. T. Burns, M. O’Brien, P. Lamperti, M. Boutillon

**Affiliations:** Bureau International des Poids et Mesures, F-92312 Sèvres Cedex, France; National Institute of Standards and Technology, Gaithersburg, MD 20899 USA; National Institute of Standards and Technology, Gaithersburg, MD 20899 USA; Bureau International des Poids et Mesures, F-92312 Sèvres Cedex, France

**Keywords:** air kerma, free-air ionization chamber, primary standard, reference radiation qualities, medium-energy x rays, x-ray calibration

## Abstract

The air-kerma standards used for the measurement of medium-energy x rays were compared at the National Institute of Standards and Technology (NIST) and at the Bureau International des Poids et Mesures (BIPM). The comparison involved a series of measurements at the BIPM and the NIST using the air-kerma standards and two NIST reference-class transfer ionization standards. Reference beam qualities in the range from 60 kV to 300 kV were used. The results show the standards to be in agreement within the combined standard uncertainty of the comparison of 0.35 %.

## 1. Introduction

An indirect comparison was made between the air-kerma primary standards for medium-energy x rays at the National Institute of Standards and Technology (NIST) and at the Bureau International des Poids et Mesures (BIPM). The measurements were conducted in March of 1991 at the BIPM and at the NIST. The measurements at the NIST were made using tungsten reference radiation qualities in the range from 60 kV to 300 kV. The reference radiation qualities used at the BIPM are those in the range from 100 kV to 250 kV recommended by the Consultative Committee for Ionizing Radiation (CCEMRI) [1]. Two NIST reference-class ionization chambers were shipped to the BIPM for the comparison.

## 2. Determination of the Air-Kerma Rate

For a free-air ionization chamber standard with measuring volume *V*, the air-kerma rate is determined by the relation

$$\dot{K}=\frac{I}{\rho_{\text{air}}V}\frac{W_{\text{air}}}{e}\frac{1}{1-g_{\text{air}}}\prod_{i}k_{i},$$ (1)

where

*I*/*ρ*_air_ *V* is the mass ionization current measured by the standard,
*W*_air_ is the mean energy expended by an electron of charge e to produce an ion pair in dry air,
*g*_air_ is the fraction of the initial electron energy lost by bremsstrahlung production in air, and
∏ *k_i_* is the product of the correction factors to be applied to the standard.

The values for the physical constants used in the determination of the air-kerma rate are given in Table 1.

## 3. Characteristics of Chambers

### 3.1 Description of Air-Kerma Standards

Both air-kerma standard chambers used in the comparison are parallel-plate free-air ionization chambers. The measuring volume *V* is defined by the diameter of the chamber aperture and the length of the collecting plate. The BIPM air-kerma standard is described in Refs. [2] and [3]. The NIST Wyckoff-Attix chamber is described in Refs. [4] and [5]. The main dimensions, the measuring volume, and the polarizing voltage for each chamber are given in Table 2.

### 3.2 Description of Transfer Ionization Chambers

Two NIST transfer chambers (NIST-T1 and NIST-T2, serial numbers 2022 and 2023 respectively) were used for the comparison. Both of the NIST transfer standards are spherical and constructed with a wall of air-equivalent plastic. Each has a nominal volume of 3.6 cm^3^, an external diameter of 1.9 cm, and a wall thickness of 0.25 mm. A collecting voltage of 300 V was applied to the NIST transfer standards. A negative polarity was used at NIST and both polarities were used at the BIPM. Both of the NIST transfer chambers were calibrated using the NIST and the BIPM standards. The calibration factors for both chambers were determined at the NIST before and after the calibrations at the BIPM.

## 4. Comparison Details

### 4.1 Irradiation Facilities and Reference Beam Qualities

The BIPM portion of the comparison was conducted at the BIPM medium-energy x-ray laboratory, which houses a constant-potential generator (maximum usable generating potential 250 kV) and a tungsten-anode x-ray tube with an inherent filtration of around 2.3 mm of aluminium. Both the generating potential and the tube current are stabilized using feedback systems constructed at the BIPM, resulting in high stability, eliminating the need for a transmission current monitor. For a well-behaved transfer instrument, the variation in the measured ionization current over the duration of a comparison is typically less than 0.04 %. The BIPM radiation qualities, which range in energy between 100 kV and 250 kV, are recommended by CCEMRI [1] and are given in Table 3.

The NIST portion of the comparison was accomplished through the use of the Wyckoff-Attix chamber in the NIST 300 kV calibration facility. The x-ray source at the time of the comparison was a 320 kV x-ray generator with a metal-ceramic x-ray tube, both supplied by Seifert^1^. The x-ray generator was a high-frequency (500 Hz), highly stabilized voltage source with an x-ray output variation of no more than ± 0.5 % in 1 h. The x-ray tube had a beryllium window of thickness 3 mm and a projected focal spot size of 5 mm^2^. The materials used for the filtration and for the measurement of half-value layer (HVL) were 99.99 % pure with thicknesses known to within ±0.01 mm. Output stability was monitored using a transmission ionization chamber. The measured currents were normalized to account for fluctuations. Used in this relative way, the absolute response of the transmission monitor does not change calibration results. The reference radiation qualities used at NIST were generated between 60 kV to 300 kV and are listed in Table 3.

### 4.2 Correction Factors

Although free-air chambers are designed to minimize or eliminate corrections to the measured ionization current, certain corrections are unavoidable. The correction factors applied to each free-air chamber and the associated uncertainties are listed in Tables 4 and 5 by radiation quality.

A correction must be made for the attenuation of the x-ray fluence along the air path *L* between the reference plane and the center of the collecting volume. The correction factor *k*_a_ is calculated using the measured or calculated air-attenuation coefficients *µ*_air_, according to

$$k_{a}=\exp(\mu_{\text{air}}L)$$ (2)

The effective attenuation path *L* varies with the temperature and pressure of the air in the chamber. The values for *k*_a_ are corrected for this effect. All ionization measurements are also corrected for the temperature and pressure of the ambient air between the radiation source and the reference plane. All measured ionization currents using the free-air chamber standards are corrected for ion recombination, *k*_s_. The ionization currents measured with the transfer standards are not corrected for ion recombination. Since the air-kerma rates used at both facilities are low, minimal volume recombination occurs in the transfer chambers. It is assumed that the difference in the recombination effect, produced at each facility, is negligible. The standard chambers are corrected for the humidity effect *k*_h_ which is taken as 0.998 for both standards and all radiation qualities.

### 4.3 Chamber Positioning and Measurement Procedure

At the NIST and at the BIPM the transfer ionization chambers and each standard chamber remain fixed in position throughout the comparison measurements. All calibrations of the transfer chambers were preceded by measurements using the standard free-air chambers. At both laboratories alignment on the beam axis was measured to around 0.1 mm and this position was reproducible to better than 0.01 mm, as observed by an alignment telescope. No correction is applied for the radial non-uniformity of each beam. The reference plane for each chamber was positioned at 1000 mm from the radiation source at the NIST and 1200 mm at BIPM. This distance was measured to 0.03 mm and was reproducible to better than 0.01 mm. The beam diameter in the reference plane was 35 mm at the NIST and 105 mm at the BIPM.

The leakage current was measured before and after each series of ionization current measurements and a correction made based on the mean of these leakage measurements. For all measurements the leakage current was less than 10^−4^ of the ionization current. For all chambers at the NIST, six sets of two to six charge measurements were made. Each charge measurement had an integration time of 60 s. The statistical standard uncertainty of the current measurements was typically less than 0.02 % for the NIST standard and transfer chambers. At the BIPM each calibration factor was obtained from a total of 80 measurements of around 30 s performed with the BIPM standard and each transfer chamber. The statistical standard uncertainty of each calibration factor is less than 0.01 %.

The polarity correction for the transfer chambers was measured at the BIPM to be only 1.0003 for both transfer chambers. Only negative polarity was used for each transfer chamber during the calibrations at the NIST. A component of 0.03 % is included in the uncertainty of the current measurement at the NIST to account for the polarity effect.

All transfer ionization chamber current measurements were normalized to 293.15 K and 101 325 Pa. The transfer chamber currents are not adjusted for humidity. The humidity is monitored and recorded at both facilities. The BIPM laboratory humidity is maintained at 50 %. The NIST laboratory average humidity is 30 %.

## 5. Measurement Uncertainties

The uncertainties associated with the primary standards are given in Table 6. The NIST uncertainties were evaluated according to Ref. [6]. The uncertainties associated with the calibration of the transfer ionization chambers at the NIST and at the BIPM are listed in Table 7. Table 8 list the uncertainty of the comparison results, *R_K_*, which are the ratios of the calibration coefficients *N_K_* for each chamber as a function of HVL. The uncertainties of the ratios *N_K,_*_NIST_/*N_K_*_,BIPM_ take into account correlations due to common Type B uncertainties associated with the physical constants and the humidity correction. There is also significant correlation in the values used for *k*_sc_ and *k*_e_. This has been accounted for in an approximate way by halving these uncertainty components.

## 6. Results and Discussion

**Fig. 1:**
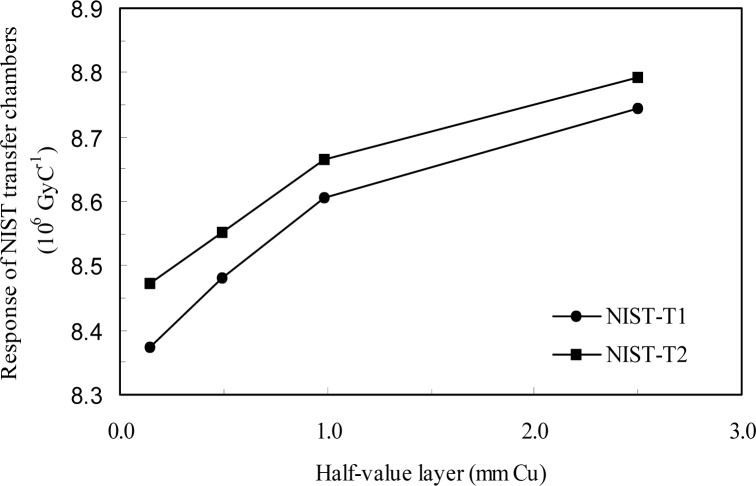
Results of the calibration of the NIST transfer chambers at the BIPM.

**Fig. 2:**
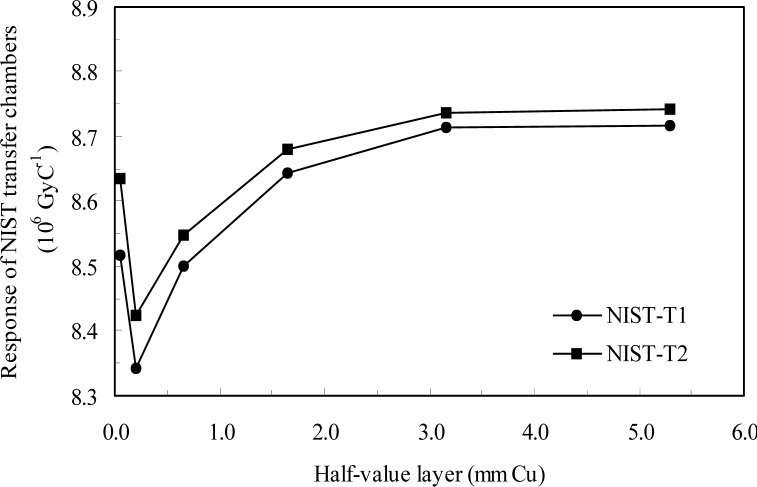
Results of the calibration of the NIST transfer chambers at the NIST.

The results for the transfer ionization chamber calibrations at both laboratories are shown in Table 9 and in Figs. 1 and 2. Since the HVL’s at the NIST and the BIPM are very different, a quartic fit was used for each transfer chamber to interpolate the NIST data in terms of HVL in order to derive comparison results *R_K_* at the HVL values which correspond to the BIPM radiation qualities;

$$R_{K}=\frac{\text{Value}\;\text{of}\;\text{fit}\;N_{K,\text{NIST}}\;\left(\text{HVL}\right)\;\text{at}\;\text{BIPM}\;\text{HVL}}{N_{K,\text{BIPM}}}.$$

A second fit using a rational function was also made. The agreement between the two fits is close, at worst 0.07 %, and an uncertainty component of 0.04 % is included in the comparison results to account for the interpolation procedure. The results, *R_K_*, of this analysis based on the quartic fits are shown in Table 10. The agreement between the two standards is within the combined standard uncertainty of the comparison of 0.35 %.

This report was not published at the completion of the analysis in June of 1992 because it was thought to be unnecessary as the findings were similar to those of the previous indirect comparison. It is now accepted practice to publish all direct and indirect comparisons at both the NIST and the BIPM. In February of 2003, a similar indirect comparison began between the BIPM and the NIST. Upon completion of the 2003 comparison, it is the intention of the authors to publish the results.

## Figures and Tables

**Table 1:** Physical constants used in the determination of the air-kerma rate

Physical constant	Value	Relative standard uncertainty (%)
*ρ*_air_^a^	1.293 kg m^−3^	0.01
*W*_air_/*e*	33.97 J C^−1^	0.15

a Density of dry air at 273.15 K and 101 325 Pa

**Table 2:** Main characteristics of the primary standards used in the comparison

Characteristic	NIST	BIPM
Air-path length (cm)	30.8	28.15
Plate separation (cm)	20.0	18.0
Collecting plate length (cm)	10.08	6.0004
Aperture diameter (cm)	0.9999	0.9939
Measuring volume (cm^3^)	7.92	4.6554
Polarizing voltage (*V*)	−5000	4000

**Table 3:** Characteristics of the reference radiation qualities used for the comparison

Reference radiation	Generating potential	Additional filtration			Half-value layer (HVL)		Air-kerma rate
	kV	mm Al	mm Cu	mm Sn	mm Al	mm Cu	mGy s^−1^
BIPM 100 kV	100	1.2032			4.027	0.148	0.21
BIPM 135 kV	135		0.2321			0.494	0.21
BIPM 180 kV	180		0.4847			0.990	0.30
BIPM 250 kV	250		1.5701			2.500	0.39
NIST M60	60	1.51			1.68	0.052	0.58
NIST M100	100	5.0			5.0	0.20	0.59
NIST M150	150	5.0	0.25		10.2	0.67	0.78
NIST M200	200	4.1	1.12		14.9	1.69	0.87
NIST M250	250	5.0	3.2		18.5	3.2	0.80
NIST M300	300	4.0		6.5	22	5.3	0.32

**Table 4:** Correction factors used in the comparison for the NIST Wyckoff-Attix standard

Correction factor	Generating potential (kV)						Relative standard uncertainty (%)	
	60	100	150	200	250	300	Type A	Type B
Air attenuation *k*_a_^a^	1.0203	1.0097	1.0068	1.0055	1.0045	1.0039		0.07
Scattered radiation *k*_sc_	0.9923	0.9940	0.9960	0.9961	0.9964	0.9968		0.07
Electron loss *k*_e_	1.000	1.000	1.0015	1.004	1.005	1.006		0.1
Ion recombination *k*_s_	1.0011	1.0011	1.0012	1.0013	1.0012	1.0009		0.1
Aperture transmission *k*_1_^b^	1.0000	1.0000	1.0000	1.0000	1.0000	1.0000		0.04
Field distortion *k*_d_	1.0015	1.0015	1.0015	1.0015	1.0015	1.0015		0.2
Polarity effect *k*_pol_	1.000	1.000	1.000	1.000	1.000	1.000	0.03	0.1
Wall transmission *k*_p_^b^	1.0000	1.0000	1.0000	1.0000	1.0000	1.0000		0.01
Bremsstrahlung 1 – *g*_air_	1.0000	1.0000	1.0000	1.0000	1.0000	1.0000		0.01

a These are nominal values for *T* = 293.15 K and *p* = 100 000 Pa. Each measurement is corrected using the air temperature and pressure measured at the time.

b The uncertainties in aperture transmission *k*_l_ and wall transmission kp are negligible.

**Table 5:** Correction factors used in the comparison for the BIPM standard

Correction factor	Generating potential (kV)				Relative standard uncertainty (%)	
	100	135	180	250	Type A	Type B
Air attenuation *k*_a_^a^	1.0102	1.0067	1.0057	1.0049	0.03	0.01
Scattered radiation *k*_sc_	0.9948	0.9962	0.9967	0.9969		0.07
Electron loss *k*_e_	1.0000	1.0023	1.0052	1.0078		0.10
Ion recombination *k*_s_	1.0004	1.0005	1.0005	1.0003	0.02	0.01
Field distortion *k*_d_	1.0000	1.0000	1.0000	1.0000		0.07
Aperture transmission *k*_l_	0.9999	0.9998	0.9997	0.9996		0.01
Wall transmission *k*_p_	1.0000	0.9999	0.9998	0.9985	0.01	
Bremsstrahlung 1 – *g*_air_	0.9999	0.9999	0.9998	0.9997		0.01

a These are nominal values for *T* = 293.15 K and *p* = 100 000 Pa. Each measurement is corrected using the air temperature and pressure measured at the time.

b The uncertainties in aperture transmission *k*_l_ and wall transmission *k*p are negligible.

**Table 6:** Relative standard uncertainties (in %) associated with the standards.

Source of uncertainty	NIST		BIPM	
	Type A	Type B	Type A	Type B
Ionization current	0.02	0.13	0.03	0.02
Volume	0.04	0.01	0.01	0.05
Positioning		0.01	0.01	0.01
Correction factors (excl. *k*_h_)	0.03	0.29	0.04	0.14
Humidity *k*_h_		0.10		0.03
Physical constants		0.15		0.15
${\dot{K}}_{\text{LAB}}$	0.05	0.36	0.05	0.21

**Table 7:** Relative standard uncertainties (in %) associated with the calibration of the transfer ionization chambers

Source of uncertainty	NIST		BIPM	
	Type A	Type B	Type A	Type B
Air kerma rate	0.05	0.36	0.05	0.21
Ionization current	0.02	0.13	0.04	0.02
Positioning		0.01	0.01	0.01

*N_K_*_,LAB_	0.05	0.38	0.07	0.21

**Table 8:** Relative standard uncertainties (in %) associated with the comparison results *R_K_*. Correlations arising from the physical constants, the humidity correction and the correction factors *k*_sc_ and *k*_e_ are removed. A type B component of 0.04 % is included to account for the use of different radiation qualities for the NIST and BIPM calibrations

Source of uncertainty	Type A		Type B
	0.09		0.3
*R_K_*		0.35	

**Table 9:** Measured results for the calibration of the NIST transfer chambers at both laboratories

Reference radiation		Half-value layer			NIST transfer chambers Calibration factors (10^6^ Gy C^−1^)	
	mm Al		mm Cu	NIST–T1		NIST–T2
					BIPM Measurements	

BIPM 100 kV	4.027		0.148	8.374		8.472
BIPM 135 kV			0.494	8.481		8.551
BIPM 180 kV			0.990	8.607		8.666
BIPM 250 kV			2.500	8.744		8.793

					NIST Measurements	

NIST M60	1.68		0.052	8.517		8.635
NIST M100	5		0.2	8.343		8.425
NIST M150	10.2		0.67	8.500		8.549
NIST M200	14.9		1.69	8.644		8.681
NIST M250	18.5		3.2	8.714		8.737
NIST M300	22		5.3	8.717		8.741

**Table 10:** Results RK of the comparison of the NIST and BIPM air-kerma standards

Generating potential (kV)	Half-value layer (mm Cu)	*R_K_* (1991)
100	0.148	0.9952
135	0.494	0.9953
180	0.990	0.9942
250	2.500	0.9930

## Footnotes

1 Certain commercial equipment, instruments, or materials are identified in this paper to foster understanding. Such identification does not imply recommendation or endorsement, nor does it imply that the equipment identified are necessarily the best available for the purpose.

## References

[1] BIPM (1972). Qualités de rayonnements.

[2] Boutillon M Mesure de l’exposition au BIPM dans le domaine des rayons X de 100 à 250 kV.

[3] Boutillon M Measuring Conditions Used for the Calibration of Ionization Chambers at the BIPM.

[4] Wyckoff HO, Attix FH (1957). Design of free-air ionization chambers. National Bureau Standards Handbook.

[5] Lamperti PJ, O’Brien M (2001). Calibration of X-Ray and Gamma-Ray Measuring Instruments.

[6] Taylor BN, Kuyatt CE (1994). Guidelines for Evaluating and Expressing the Uncertainty of NIST Measurement Results.

